# EGF suppresses the expression of miR-124a in pancreatic β cell lines via ETS2 activation through the MEK and PI3K signaling pathways

**DOI:** 10.7150/ijbs.34985

**Published:** 2019-09-07

**Authors:** Lin Yang, Yuansen Zhu, Delin Kong, Jiawei Gong, Wen Yu, Yang Liang, Yuzhe Nie, Chun-Bo Teng

**Affiliations:** College of Life Science, Northeast Forestry University, Harbin, China

**Keywords:** EGFR signaling, MicroRNA-124a, ETS2, Beta cells, Insulin.

## Abstract

Diabetes mellitus is characterized by pancreatic β cell dysfunction. Previous studies have indicated that epidermal growth factor (EGF) and microRNA-124a (miR-124a) play opposite roles in insulin biosynthesis and secretion by beta cells. However, the underlying mechanisms remain poorly understood. In the present study, we demonstrated that EGF could inhibit miR-124a expression in beta cell lines through downstream signaling pathways, including mitogen-activated protein kinase kinase (MEK) and phosphatidylinositol 3-kinase (PI3K) cascades. Further, the transcription factor ETS2, a member of the ETS (E26 transformation-specific) family, was identified to be responsible for the EGF-mediated suppression of miR-124a expression, which was dependent on ETS2 phosphorylation at threonine 72. Activation of ETS2 decreased miR-124a promoter transcriptional activity through the putative conserved binding sites AGGAANA/TN in three miR-124a promoters located in different chromosomes. Of note, ETS2 played a positive role in regulating beta cell function-related genes, including miR-124a targets, Forkhead box a2 (FOXA2) and Neurogenic differentiation 1 (NEUROD1), which may have partly been through the inhibition of miR-124 expression. Knockdown and overexpression of ETS2 led to the prevention and promotion of insulin biosynthesis respectively, while barely affecting the secretion ability. These results suggest that EGF may induce the activation of ETS2 to inhibit miR-124a expression to maintain proper beta cell functions and that ETS2, as a novel regulator of insulin production, is a potential therapeutic target for diabetes mellitus treatment.

## Introduction

Diabetes mellitus, including type 1 (T1D) and type 2 diabetes (T2D), is a prevalent disease worldwide. A better understanding of the biological and physiological role of pancreatic β cells could help develop new therapeutic strategies 1-4. MiRNAs have been indicated to play an important role in human islet β cell biology and to be potential candidates for a new pharmacological strategy 5-8. Initially described as a brain‐specific miRNA in mammals 9, 10, MiR-124a-3p (miR-124a), is also well represented in the mouse pancreatic MIN6 β‐cell line 11. Increasing expression levels of miR-124a have been found during embryonic pancreas development 12 and differentiation of human induced pluripotent stem cells (hiPSCs) into insulin-producing cells 13, indicating its function in pancreatic β cell development. Emerging data suggest that miR-124a is overexpressed in pancreatic islets in humans with T2D compared to that in subjects without diabetes 14-16. MiR-124a has also been shown to play a negative role in insulin biosynthesis and secretion by directly targeting the expression of the glucose-sensing regulator forkhead box a2 (FOXA2) 12, 15, and some key components of the exocytotic machinery, such as Rab27 17, NOC2 17, and MTPN 15 in pancreatic β cells. NeuroD1, a transcription factor involved in insulin gene transcription 18 was also predicted to be the targets of miR-124a 19, and was increased in mRNA levels upon miR-124a silencing 15. These studies indicate that miR-124a is potentially involved in beta cell dysfunction in T2D. Furthermore, the low expression levels of miR-124a in pancreatic islets in subjects without diabetes (in comparison with several other pancreatic islet microRNAs) 14, 20, suggests the need to maintain low miR-124a expression levels in adult insulin-producing cells for proper function.

Epidermal growth factor (EGF) has a complex role in pancreatic development and beta cell maintenance and function. EGF binds to its receptor EGFR for signaling via multiple downstream pathways, including PI3K/AKT, Ras/MEK/ERK1/2, JNK, JAK/STAT3, and others 21, 22. EGFR signaling is required for normal beta cell development, as EGFR-/- mice display impaired beta cell mass and disrupted islet migration 23, and because constitutively active EGFR (L858R) increases beta cell proliferation and mass in newborn mice 24. In addition, a recent study has shown that EGF-mediated EGFR signaling could inhibit fetal beta cell differentiation via apical polarity 25. EGFR signaling is also essential for beta cell proliferation after birth, and even a modest attenuation of this pathway leads to severe defects in the postnatal growth of beta cells 26. EGFR is also involved in beta cell proliferation and survival in response to high-fat diet, pregnancy 27 and pancreatic duct ligation 28, as well as other extracellular stimuli that induce compensatory expansion of beta cells 24, 29, 30. Interestingly, in the adult pancreas, EGF plays a role in acinar to beta cell reprogramming in mice with chronic hyperglycemia 31, and it is indispensable for the in vitro generation of insulin-producing beta cells from adult pancreatic acinar cells or human pluripotent stem cells 32-34. Additionally, there are studies showing that EGF can stimulate insulin secretion by activating phospholipase D2 (PLD2) 35, 36. Our previous studies have shown that EGFR signaling can inhibit miR-124a expression to regulate pancreatic progenitor cell proliferation 37. Therefore, we wondered whether there exists a similar mechanism of EGFR signaling modulating miR-124a expression for proper beta cell activity.

In this study, we investigated the regulatory effect of EGFR signaling on miR-124a in pancreatic beta cells. We found that EGF suppressed miR-124a expression in beta cell lines, which was mainly attributed to the ERK and PI3K pathways downstream of EGFR signaling cascades. We further identified that the activation of ETS2, an ETS (E26 transformation-specific) transcription factor, contributed to the inhibition of miR-124a expression by EGF. The putative ETS2-binding sites on the miR-124a promoters were also determined. In addition, we demonstrated the positive effects of ETS2 on insulin biosynthesis.

## Materials and Methods

### Cell culture

The murine pancreatic β cells MIN6, obtained from ATCC (Manassas, VA, USA), were cultured in High-Glucose DMEM (HyClone™, Logan, UT) containing 15% (v/v) FBS (Biological Industries, Kibbutz Beit Haemek, Israel), 1% (v/v) GlutaMax Supplement (Gibco, Grand Island, NY, USA), 1% (v/v) penicillin-streptomycin (HyClone™, Logan, UT), and 1× B27 (Invitrogen, Carlsbad, CA) at 37°C in a 5% CO2 atmosphere. The rat cell line INS-1 was maintained in RPMI‐1640 medium (HyClone™, Logan, UT) containing 10% FBS, 1% GlutaMax Supplement, 1% penicillin-streptomycin, 50 μmol/L 2‐mercaptoethanol (Sigma, St. Louis, MO) and 1 mM sodium pyruvate (HyClone™, Logan, UT).

### Analysis of gene expression

RNA was extracted with Tripure™ reagent (Roche, Basel, Switzerland), and cDNA was synthesized with TransScript II One-Step gDNA Removal and cDNA Synthesis SuperMix (TransGen Biotech, Beijing, China). Mature miR-124a was quantified based on the stem-loop reverse transcript qPCR method using the artificially designed stem-loop-RT primer and qPCR primers. MiR-124a expression was normalized to snRNA U6 (*RnU6*). Meanwhile, cDNA transcribed through oligo (dT) was used for the analysis of other gene expression, and *ACTB* (*β-actin*) was used as an internal control. qPCR was performed using SYBR Green Mix (Roche, Indianapolis, USA) with a Roche LightCycler 480 II Real-Time PCR System. The primer sequences used here are listed Tables S1 and S2 in supplementary materials.

### Transient transfection

Small interfering RNAs (siRNAs) and miRNA mimics were designed and synthesized by GenePharma (Shanghai, China, Table S4). Plasmids were extracted and purified using the EasyPure HiPure Plasmid MiniPrep Kit (TransGen Biotech, Beijing, China). Cells were transfected with RNA oligonucleotides or plasmids using Lipofectamine RNAiMAX or 2000 (Thermo Fisher Scientific, Wilmington, USA) according to the manufacturer's instructions. After 24 hours, the cells were treated with or without DMSO (Sigma, St. Louis, MO), 20 μM U0126 (Beyotime, Shanghai, China), 20 μM LY294002 (Beyotime, Shanghai, China), or 5 μM Gefitinib (Sigma, St. Louis, MO). At 36 to 48 hours posttransfection, the cells were harvested for real-time qPCR or Western blotting. For EGF (R&D Systems, Minneapolis, USA) treatment, 24 hours posttransfection, cells were starved for 12 hours and 20 ng/mL EGF was added for another 12 h. Then, cells were harvested for real-time qPCR or Western blotting.

### Plasmids construction

The full-length mouse ETS2 gene (NM_011809.3) was amplified using cDNA from MIN6 cells, and the fragment was cloned into the overexpression vector pcDNA3.1 (+) using One Step Cloning Kit II (Vazyme, Nanjing, China). The ETS2-T72A mutant plasmid was constructed using artificially designed primers and the ClonExpress MultiS One Step Cloning Kit (Vazyme, Nanjing, China). The miR-124 promoter region (-2000 to +1) containing the ETS2-binding motif was amplified by PCR from MIN6 cells. The fragments were cloned into the pGL3-Basic luciferase reporter vector (Promega, Madison, USA), and mutations in the ETS2-binding site were created through multiple pairs of primers and then cloned into pGL3-Basic vector via the ClonExpress MultiS One Step Cloning Kit (Vazyme, Nanjing, China). Primers are listed in Table S3 in supplementary materials.

### Western blotting

Cells in 6-well dishes were lysed in RIPA buffer (Beyotime Biotechnology, Shanghai, China) containing protease and phosphatase inhibitor cocktails (Thermo Fisher Scientific, Waltham, MA, USA) and incubated on ice for 15 min. The resulting lysate was centrifuged at 13,000 rpm for 10 min at 4°C. Aliquots of the supernatant were removed for protein quantification using the BCA (bicinchoninic acid assay) kit (Beyotime, Shanghai, China). An equal volume of 5x SDS sample buffer (125 mM Tris-hydrochloric acid, pH 6.8, 4% SDS, 4% 2-mercaptoethanol, and 15% glycerol) was added to each cell lysate sample and incubated for 10 min at 95°C. Equal amounts of protein were loaded on 12% polyacrylamide gels, electrophoresed, and transferred to nitrocellulose filter membranes (Millipore, Billerica, MA, USA). After blocking the membranes with 5% skim milk for 30 min, primary antibodies were reacted with target antigens at 4°C overnight. Secondary antibodies (horseradish peroxidase-conjugated anti-mouse IgG or anti-rabbit IgG) were then incubated with the membranes for 1 h at room temperature. Immunoreactive bands were detected with an ECL Detection Reagent (Tanon, Shanghai, China). Protein levels were normalized to total protein concentrations. The primary antibodies used in this study were against the following: AKT (cat# 4691), p-AKT 473 (cat# 4060), p-AKT 308 (cat# 2965), ERK1/2 (cat# 9102), and p-ERK1/2 (cat# 9101), from Cell Signaling Technology (MA, USA); p-ETS2 (Thr72) (cat# 44-1105G, Thermo Fisher Scientific, Wilmington, USA), ETS2 (cat# ab230519, Abcam, Cambridge, UK), Neurod1 (cat# 12081-1-AP, Proteintech, Chicago, USA), FoxA2 (cat# 22474-1-AP, Proteintech, Chicago, USA), and PDX1 (cat# ab47383, Abcam, Cambridge UK).

### Dual-luciferase reporter assay

MIN6 cells were transfected with promoter reporter plasmids and the internal control luciferase vector pRL-TK. Meanwhile, ETS2 overexpression vector or empty vector pCDNA3.1(+) was cotransfected into cells using Lipofectamine 2000 reagent (Invitrogen, California, USA). Forty-eight hours later, cells were lysed, and luciferase activities were measured using a Dual-Luciferase Reporter Assay System (Promega, Madison, USA) on a GloMax^TM^ 20/20 Luminometer (Promega, Sunnyvale, USA) according to the manufacturer's instructions. For analysis, the firefly luciferase activity was normalized to the Renilla luciferase activity.

### Immunofluorescence

After 72 h of transfection, cells were gently washed with 0.01 M PBS (phosphate-buffered saline, pH 7.4), fixed with 4% paraformaldehyde (in PBS) for 20 min at room temperature and then permeabilized with 0.3% Triton X-100 (in PBS) for 13 to 17 min. After blocking with 10% horse serum for 50 min at 37℃, cells were incubated with a primary antibody against PDX1 (cat# ab47383, Abcam, Cambridge UK) or anti-C-peptide (cat# 4593s, Cell Signaling Technology, MA, USA), which had been diluted in 10% horse serum (in PBS), overnight at 4℃. Next, the cells were incubated with secondary antibody diluted in 1% BSA for an hour at 37℃. Cell nuclei were stained with Hoechst 33342 (cat# C1022, Beyotime, Shanghai, China) for 5 min at room temperature. Images were captured using DeltaVision OMX (General Electric Company, Fairfield, Connecticut, USA).

### Islets isolation and culture

According to the collagenase digestion method 38, islets were isolated from 8-week-old C57BL/6 female mice with similar weights, which were purchased from the Second Affiliated Hospital of Harbin Medical University (China). Freshly isolated islets were handpicked for purification, and cultured in RPMI 1640 plus 10% FBS and 1× B27 for 3 h before transfected with lentiviral particles. After 24 h of infection, the viral supernatant was replaced by fresh media. 72 h post-infection, they were counted for RNA or protein extraction (200 islets per group) or insulin measurement (10 islets per group). All animal procedures were performed according to protocols approved by the Animal Care and Use Committee of Northeast Forestry University.

### Lentivirus preparation

Lentiviral plasmid of shETS2 was constructed through annealing the two primers (Table S3) into pLKO.1-EGFP-puro vector which was cutted with *EcoRI* and *AgeI*. Lentivirus was prepared in a polyethylenimine-based method 39. Briefly, 293T cells were co-transfected with the packaging plasmids pPAX2, pcDNA-MDG, and the transfer plasmids pLKO.1-EGFP-Puro-shETS2-3153 by polyethylenimine for 48 h before harvesting lentiviral particles from the culture medium.

### Cellular insulin content measurement and glucose-stimulated insulin secretion assay

One day before transfection, MIN6 cells were seeded in 24-well cell culture plates and then transfected with ETS2 siRNAs or ETS2 expression plasmids or control for 48 h as described above, and the medium was then replaced with DMEM-based complete growth medium (containing 3 mM glucose) 16 h prior to experiments. Next, the cells (or islets infected with shNC or shETS2 lentiviral particles for 72 h) were incubated with glucose-free Krebs-Ringer bicarbonate (KRB) HEPES buffer (115 mM NaCl, 4.7 mM KCl, 1.2 mM MgCl2, 1.2 mM KH2PO4, 20 mM NaHCO3, 16 mM HEPES, 2.56 mM CaCl2, and 0.2% BSA) for 1 h. Then, the cells were collected and homogenized by sonication in phosphate-buffered saline (PBS), while islets were lysed in PBS using a 27-gauge syringe and homogenized by sonication. The cellular insulin content was measured according to a mouse insulin ELISA kit (Njjcbio, Nanjing, China). For the glucose-stimulated insulin secretion assay, after incubation for 1 h in glucose-free KRBH buffer, the cells or islets were treated for 1 hour in KRBH buffer with low (3 mM for MIN6 cells, 2.8 mM for islets) or stimulatory (20 mM for MIN6 cells, 16.7 mmol/l for islets) concentrations of glucose. Supernatants were obtained for the determination of insulin concentration using an ELISA kit. Data were normalized to total protein content, which was measured using a bicinchoninic acid assay.

### Statistical analysis

Analysis was performed with GraphPad Prism v.7.0 (GraphPad Software Inc., San Diego, CA, USA). Values were expressed as the mean ± standard error of the mean (SEM) of at least three independent experiments. Significant differences were determined using unpaired two-tailed Student's t-test for two-group comparisons or one-way ANOVA for multiple comparisons. P < 0.05 was considered statistically significant.

## Results

### EGF inhibits miR-124a expression in both MIN6 and INS-1 cell lines

**Figure 1:**
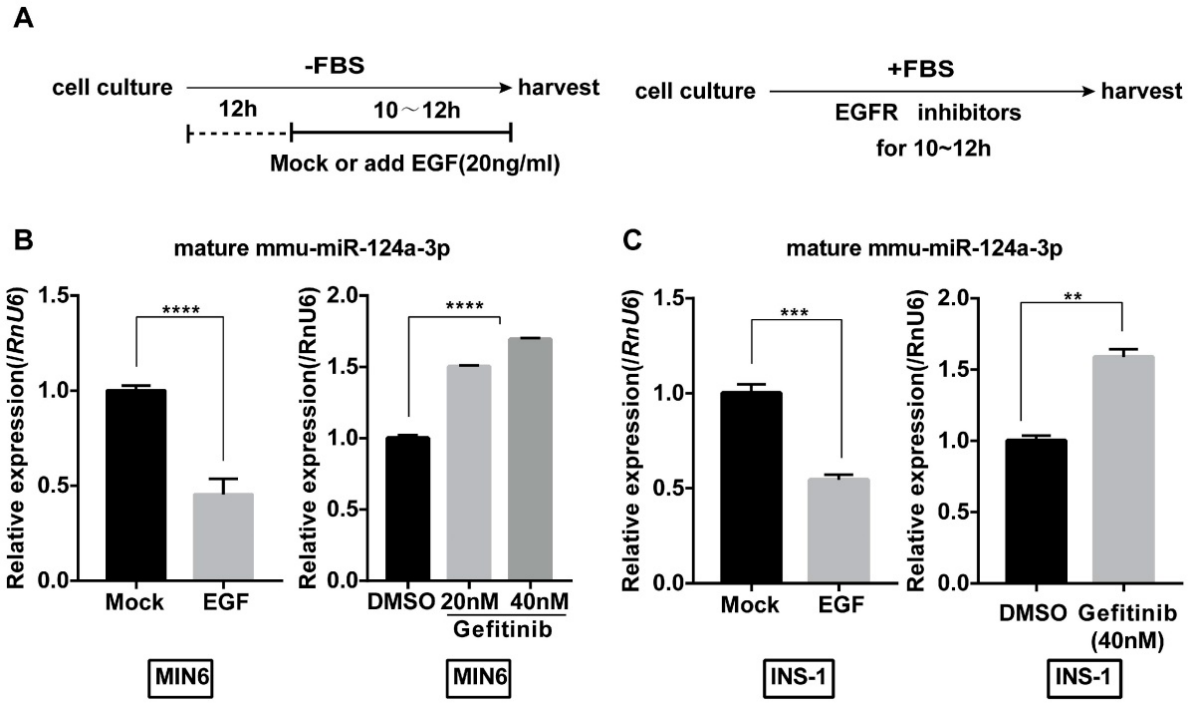
** MiR124a expression is regulated by EGFR signaling in insulinoma cells.** (A) Schematic diagram for EGF or inhibitor treatment is displayed. For growth factor treatment (left), cells were first serum-starved for 12 hours (h) and then incubated with serum free of EGF (20 ng/mL) or not for the indicated times, while inhibitors were added without the serum starvation step (right). (B) qRT-PCR was used to determine the expression of mature miR-124a after treatment with EGF (left) or the EGFR inhibitor gefitinib at the indicated concentration (right) in MIN6 cells. (C) The same as in (B), but in INS1 cells. *RnU6*, U6 small nuclear RNA, internal control for miRNA detection. Data represent the mean ± SE of three independent experiments (each n = 3). * P < 0.05, ** P < 0.01, *** P < 0.001, **** P < 0.0001.

We first examined the effect of EGF on the expression of miR-124a in MIN6 cells. To eliminate the effect of EGF in serum, we starved MIN6 cells for 12 hours in serum-free medium and then treated them with or without EGF (20 ng/mL) under serum-free conditions (Fig. 1A). Levels of mature miR-124a in MIN6 cells started to decrease 6 hours after EGF treatment (Fig. 1B). Moreover, we found that gefitinib, an EGF receptor inhibitor, significantly upregulated the levels of miR-124a to a limited scale in a concentration-dependent manner (Fig. 1B). Interestingly, we also found the same effect of EGF and gefitinib in another β cell line, the rat cell line INS-1 (Fig. 1C). These results indicate that EGF could inhibit miR-124a expression in beta cells and that this pattern of regulation may be conserved among species to some extent.

### EGF inhibits miR-124 expression by activating ERK and AKT signaling cascades

**Figure 2:**
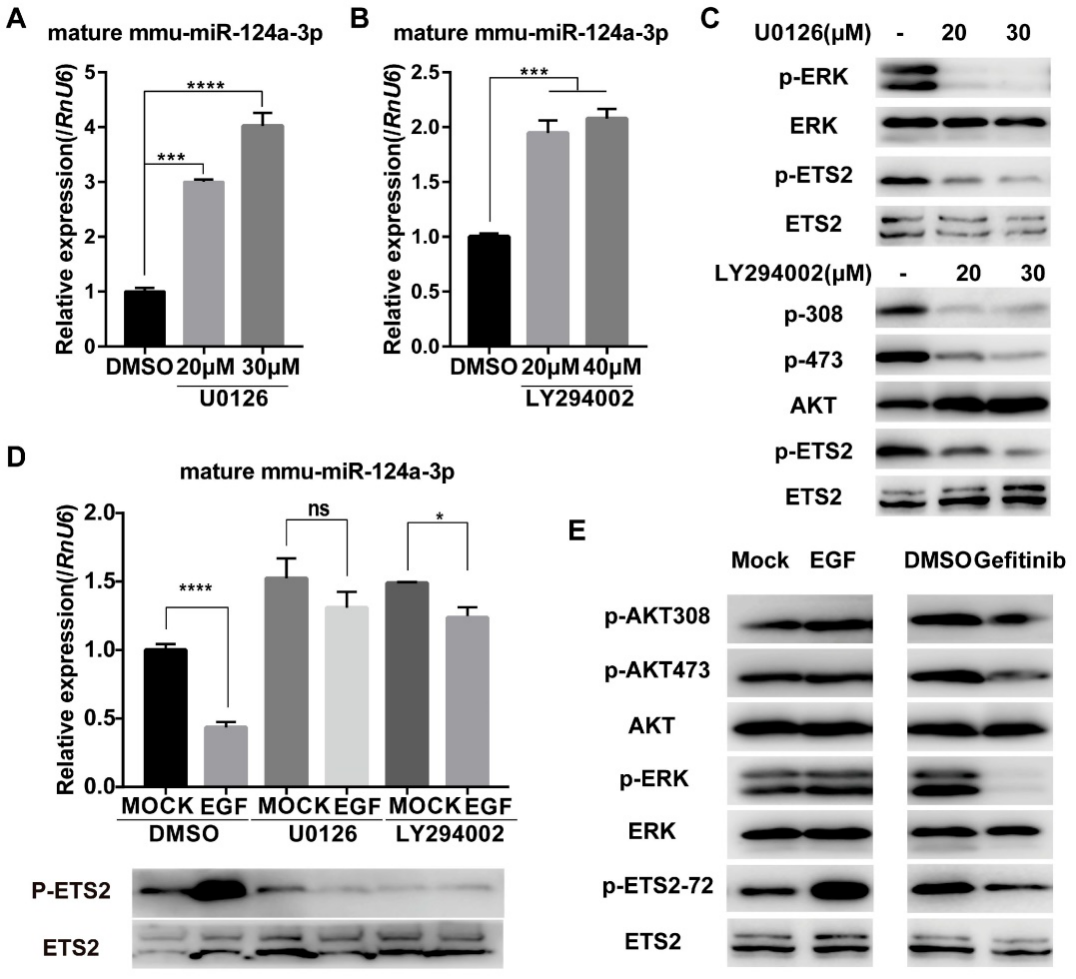
** Ras/ERK and PI3K/AKT signaling pathways control miR-124a expression.** (A) Expression of miR-124a was determined by qRT-PCR in MIN6 cells treated with the inhibitor to MEK (U0126) at the indicated concentration or DMSO for 12 h. (B) The same as in (A), but in cells treated with the inhibitor to PI3K (LY294002) or DMSO. (C) Top: Western blotting was used to monitor the expression of phosphorylated ERK, total ERK, phosphorylated ETS2 (Thr72) and total ETS2 in the presence or absence of U0126; bottom: Western blotting for the levels of phosphorylated AKT (Ser308 and Thr473), total AKT, phosphorylated ETS2 (Thr72) and total ETS2 in the presence or absence of LY294002. (D) qRT-PCR (top) for the expression of miR-124a in MIN6 cells, which were serum-starved overnight and then treated with U0126, LY294002 or DMSO for 30 min prior to the addition of EGF; Western blot for protein levels of phosphorylated ETS2 (Thr72) and total ETS2 in cells under the same conditions as above. (E) Western blotting was used to monitor the expression of phosphorylated AKT, total AKT, phosphorylated ERK, total ERK, phosphorylated ETS2 (Thr72) and total ETS2 in MIN6 cells treated with EGF (left), gefitinib (right) or control medium. *RnU6*, U6 small nuclear RNA, internal control for microRNA detection. *ACTB*, actin gene, internal control for cDNA detection. ACTB, actin, internal control for protein detection. Data represent the mean ± SE of three independent experiments (each n = 3). * P < 0.05, ** P < 0.01, *** P < 0.001, **** P < 0.0001. ns, not significant.

EGF-EGFR is well known to stimulate the PI3K/AKT and ERK1/2 signaling pathways 21, 22. To determine whether these pathways are required for EGF-induced suppression of miR-124a expression, we first treated MIN6 cells with specific inhibitors of these pathways, including U0126 (MEK inhibitor) and LY294002 (PI3K inhibitor). U0126 suppressed the phosphorylation of ERK1/2 in MIN6 cells (Fig. 2C) and was found to stimulate miR-124a expression (Fig. 2A). Additionally, treatment with LY294002 resulted in a similar increase in the level of miR-124a (Fig. 2B) and a decrease in the phosphorylation of AKT at Ser473 (Fig. 2C). Furthermore, both U0126 and LY294002 nearly abrogated the EGF-stimulated decrease in miR-124a expression (Fig. 2D). These observations suggest that the ERK members of the MAPK family and PI3K pathway played an essential role in miR-124a expression in β cells in response to EGF.

We proposed that EGF may inhibit miR-124a transcriptionally by activating some transcription factors that are downstream effectors of the EGFR signaling pathway and can associate with the miR-124a promoter. The well-defined nuclear targets of the Ras/MAPK pathway include members of the ETS family of transcription factors, including ELK1 and ETS2 40. Studies have shown that Ras can stimulate ETS2 phosphorylation at threonine 72 through the Raf/MAPK pathway 41, 42 and some studies have shown that the PI3K/AKT pathway can also lead to the phosphorylation of ETS2 43, resulting in the ability of ETS2 to activate or repress specific target genes. A decrease in ETS2 phosphorylation at threonine 72 was observed after the treatment of MIN6 cells with the MEK or PI3K inhibitor (Fig. 2C). We also confirmed that EGF could stimulate ETS2 phosphorylation at threonine 72, accompanied by a marked increase in MAPK phosphorylation and a slight increase in AKT phosphorylation in MIN6 cells. The EGFR inhibitor affected ETS2 phosphorylation by abrogating ERK1/2 phosphorylation with slight dephosphorylation of AKT (Fig. 2E). In addition, miR-124a levels were negatively correlated with ETS2 phosphorylation after U0126 and LY294002 treatment prior to EGF incubation (Fig. 2D). Therefore, we speculate that ETS2 was one of the targets downstream of the EGF-MAPK/PI3K axis, leading to the downregulation of miR-124a expression.

### ETS2 represses the expression of miR-124a dependent on ERK- or/and AKT- mediated phosphorylation

**Figure 3:**
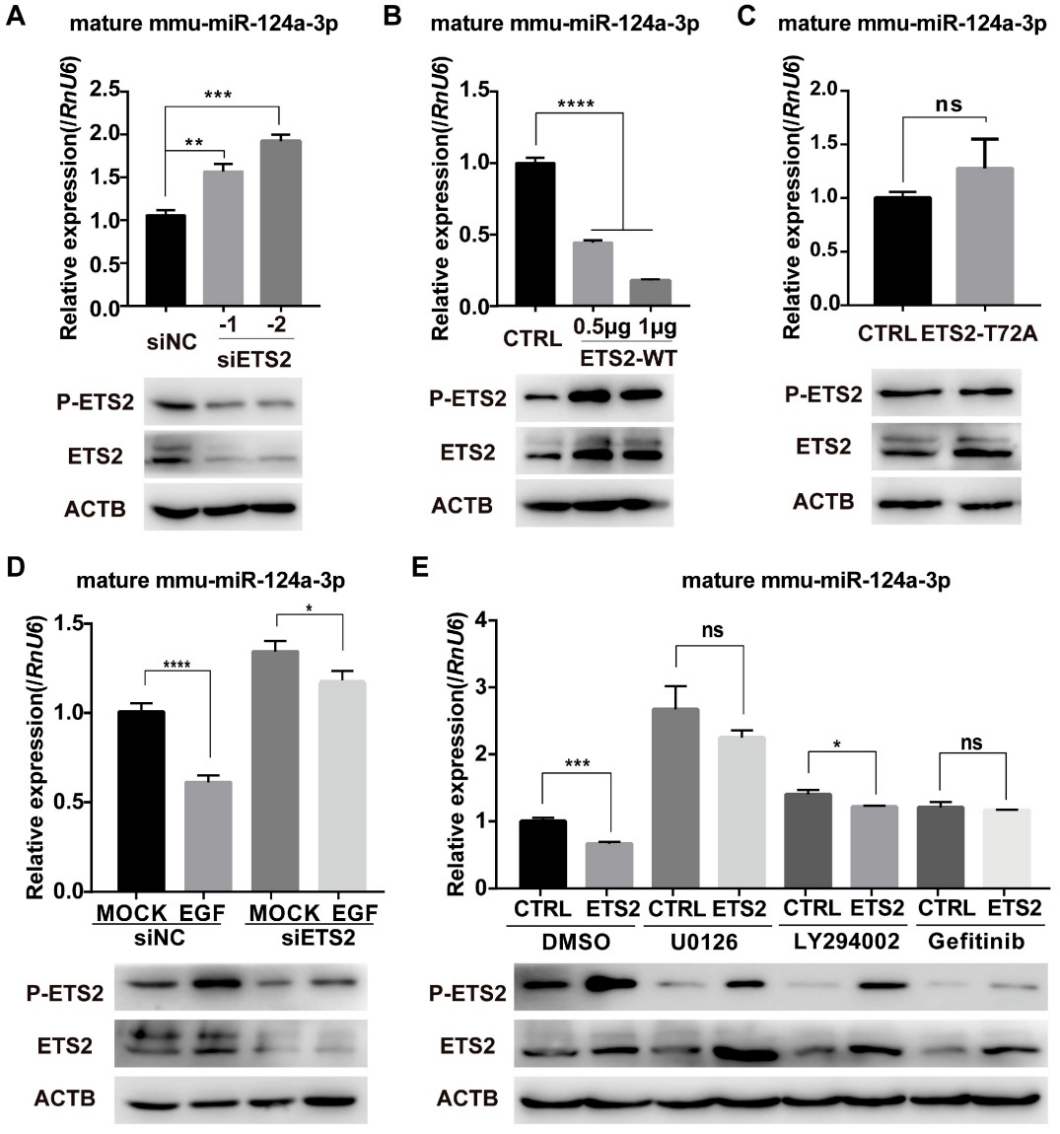
** Phosphorylated ETS2 affects miR-124a expression.** (A) Expression of ETS2 (phosphorylated and total) and mature miR-124a was determined in MIN6 cells by Western blotting and qRT-PCR, respectively, after transfection with siRNAs targeting ETS2 (siETS2, 50nM) or negative control (siNC) for 48 h. (B) The same as in (A), but in cells transfected with the indicated amount of wild-type ETS2 overexpression vector (ETS2-WT) or empty vector for 48 h. (C) The same as in (B), but in cells transfected with the overexpression vector of mutant ETS2, whose phosphorylation site at threonine 72 was replaced to alanine (ETS2-T72A), or empty vector. (D) Expression of mature miR-124a was determined by qRT-PCR (top) in MIN6 cells transfected first with siETS2 or siNC for 36 h and subsequently serum-starved for 12 h before EGF treatment. Expression of phosphorylated and total ETS2 was monitored by Western blotting (bottom) under the same conditions. (E) Expression of mature miR-124a and ETS2 (phosphorylated and total) was determined by qRT-PCR (top) and immunoblotting (bottom), respectively, in MIN6 cells transfected first with vector containing the wide-type *ETS2* coding sequence or control vector for 48 h and subsequently treated with DMSO, U0126, LY294002, or gefitinib. Data represent the mean ± SE of three independent experiments (each n = 3). * P < 0.05, ** P < 0.01, *** P < 0.001, **** P < 0.0001. ns, not significant.

To further determine the role of ETS2 in EGF-induced inhibition of miR-124a expression, we transfected MIN6 cells with ETS2-targeting siRNAs or NC (negative control, scrambled sequences), and an increase in miR-124a expression was observed 48 h post-transfection (Fig. 3A), while ETS2 overexpression indeed blocked the expression of miR-124a as expected (Fig. 3B). In addition, when the mutant type of ETS2 (with the phosphorylation site at threonine 72 replaced by alanine) was overexpressed, no change in miR-124a expression was observed (Fig. 3C). We also examined miR-124a expression levels in cells in which ETS2 was knocked down by siRNAs before EGF treatment. Pretreatment of MIN6 cells with siETS2 was found to eliminate the EGF-stimulated inhibition of miR-124a expression (Fig. 3D), indicating that ETS2 was essential for this inhibition. Moreover, the ETS2 overexpression-induced decrease in miR-124a expression was reversed by inhibitors of MEK, PI3K and EGFR (Fig. 3E), which are important for EGF-induced ETS2 activation, as shown in Fig. 2. Additionally, a negative correlation between ETS2 phosphorylation and miR-124a expression was observed (Fig. 3D, 3E). These results indicated that ETS2 participated in the regulation of miR-124a levels, and its negative role in this process was dependent on its phosphorylation at threonine 72, which was activated by EGF-induced MAPK or/and PI3K signaling pathways. Since other growth factors and related stimuli could stimulate the MAPK pathway-mediated phosphorylation of ETS2 44, it was not surprising that treatment with U0126 resulted in a greater increase in miR-124a expression levels than treatment with gefitinib, even with ETS2 overexpression (Fig. 3E).

### ETS2 targets the promoters of miR-124a for inhibition

**Figure 4:**
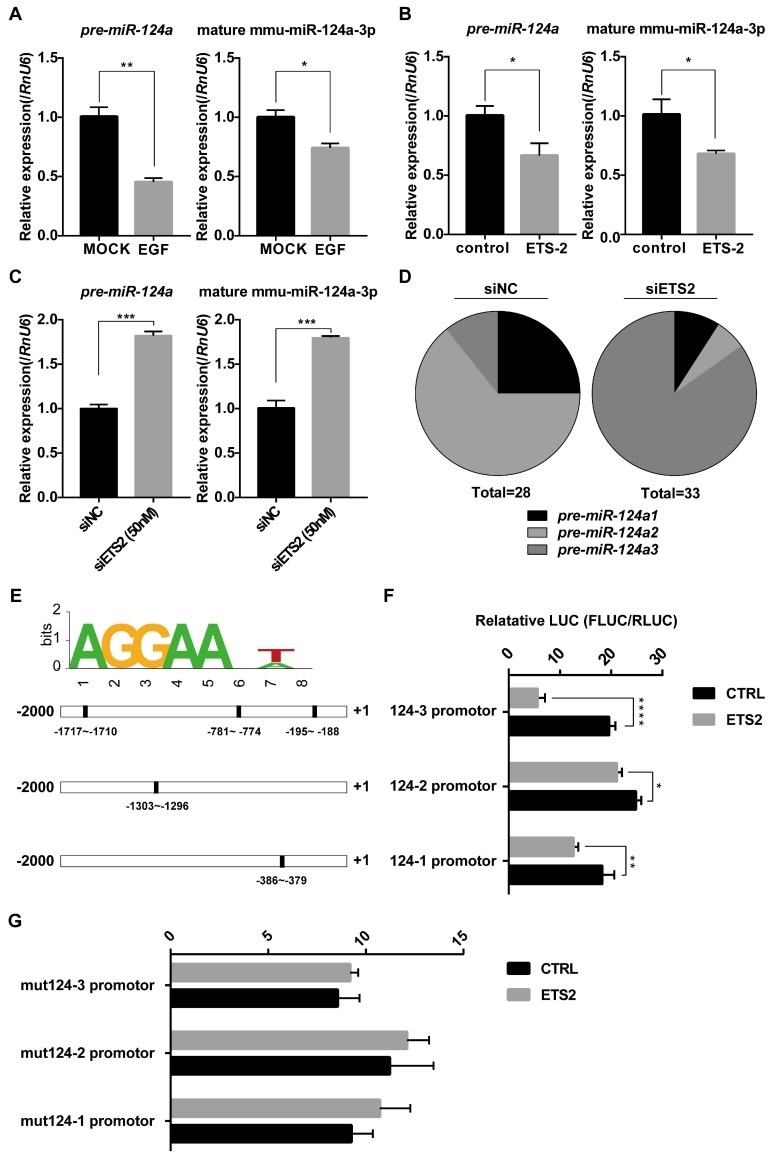
** ETS2 targets the promoters of miR-124a for inhibition.** (A) Expression of mature miR-124a and pre-miR-124a was determined by qRT-PCR in MIN6 cells under EGF treatment. (B) The same as in (A), but in MIN6 cells transfected with siETS2 or siNC. (C) The same as in (A), but in MIN6 cell transfected with ETS2 overexpression vector or control vector. (D) Proportion of *pre-miR-124a* encoded from three different genomic loci (*pre-miR-124a1*, *2*, and *3*) was analyzed by clone sequencing (as describe in material and methods) in MIN6 cells treated with siNC (left) or siETS2 (right). Total = 28 or 33 indicates the number of positive clones used in this analysis. (E) A putative ETS2-binding sequence is illustrated (top). Bottom: putative ETS2-binding elements (filled rectangle) are represented in the promoters (-2000 to +1) of miR-124a1, 2 and 3 (outlined rectangle). (F) Luciferase assays were performed to test the effect of ETS2 on miR-124a promoters 1, 2, and 3 in MIN6 cells as described in methods. FLUC: firefly luciferase activity. RLUC: Renilla luciferase activity. (G) Luciferase assays were performed to test critical binding sites for ETS2 to targeting using mutated miR-124a promoters. Mutations were introduced by deleting the first five conserved AGGAA and the seventh nucleotide A/T of the putative ETS2-binding motif. Data represent the mean ± SE of three independent experiments (each n = 3). * P < 0.05, ** P < 0.01, *** P < 0.001, **** P < 0.0001. ns, not significant.

There are three miR-124 precursor genes located on chromosomes 14, 3, and 2 in the mouse genome, encoding mature miR-124a sequences, miR-124a, miR-124a2, and miR-124a3, respectively. To evaluate whether the negative regulation of miR-124a by ETS2 was a direct transcriptional modulation, we first investigated pre-miR-124a expression either under EGF-treated or ETS2-overexpressing conditions or under ETS2-silenced conditions using one pair of primers simultaneously amplifying isoforms 1, 2, and 3 (as shown in Fig. S1A). Corresponding changes in pre-miR-124a expression were observed, as expected (Fig. 4A-4C), indicating that promoter control by ETS2 transcription factor may be responsible for the suppression of miR-124 expression. In addition, after ETS2 knockdown, a significant variation in the proportion of three miR-124a precursors existing in MIN6 cells was observed, and the proportion of pre-miR-124a3 was markedly increased (Fig. 4D). The miR-124a promoter was next investigated using the online analysis tool ALEEGN-PROMO 45 and the JASPAR database for the identification of putative transcription factor-binding sites. Regions 2500 bases upstream and 100 downstream from the transcription start site, which is highly conserved among human, mouse and rat genomes 9, 46, were analyzed for the miR-124a genes. Three of the putative ETS2-binding sites (AGGAANA/TN) were identified on the miR-124a3 promoter, while only one putative ETS2-binding motif was found to be located on the miR-124a1 and miR-124a2 promoters. As shown in the schematic in Fig. 4E, the positions are indicated relative to the transcription initiation site, and most of these binding sites are evolutionarily conserved between mice and rats (Fig. S1B). Subsequently, miR-124a promoter constructs fused to a luciferase reporter were cotransfected with plasmids encoding ETS2, and the results showed that the overexpression of ETS2 could repress the activity of all three miR-124a promoters, especially the miR-124a3 promoter (Fig. 4F). These data were consistent with the results showing that pre-miR-124a3 expression levels were markedly increased after ETS2 knockdown. Furthermore, when mutations were introduced into the ETS2-binding sites by deleting the AGGAA sequence and the seventh nucleotide A/T, the transrepression activity of ETS2 nearly disappeared (Fig. 4F). Together, these findings indicate that ETS2 could bind to highly conserved sites on the miR-124a promoter to induce transcriptional repression.

### ETS2 regulates Foxa2 and NeuroD1 expression partly by inhibiting miR-124a expression

**Figure 5:**
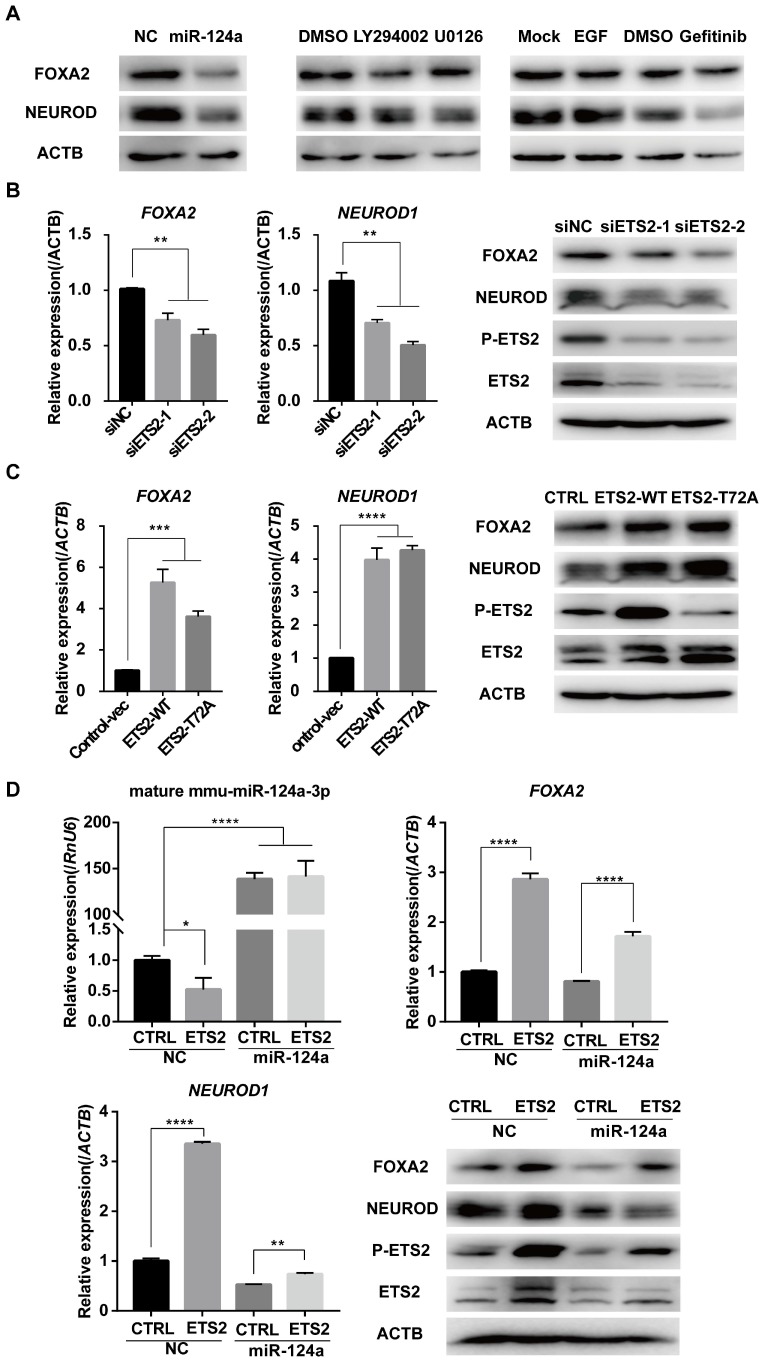
** ETS2 regulates Foxa2 and NeuroD1 expression partly by inhibiting miR-124a expression.** (A) Expression of FOXA2 and NEUROD1 was determined by immunoblotting in MIN6 cells transfected with negative control (NC) or miR-124a mimics, or incubated with EGF or not, or incubated with DMSO, LY294002, U0126 or gefitinib. (B) Expression of *FOXA2* and *NEUROD1* were determined by qRT-PCR in MIN6 cells following siETS2 or siNC transfection for 72 h. Subsequently, Western blotting was used to monitor the protein expression of FOXA2, NEUROD, phosphorylated ETS2 (Thr72) and total ETS2. ACTB (β-actin) was used as a loading control. (C) The same as in (B), but in MIN6 cells transfected with plasmids overexpressing wide-type (ETS2-WT) or mutant ETS2 (ETS2-T72A) or empty vector for control. (D) Expression of miR-124a, *FOXA2* and *NEUROD1* were determined by qRT-PCR and western blot in ETS2-overexpression or control MIN6 cells following miR-124a mimics or NC transfection. To restore miR-124a expression, MIN6 cells were co-transfected with the indicated combinations of mimics and vectors for 72 h. *ACTB*, the actin gene, internal control for cDNA detection. ACTB, actin, internal control for protein detection. Data represent the mean ± SE of three independent experiments (each n = 3). * P < 0.05, ** P < 0.01, *** P < 0.001, **** P < 0.0001.

Several reports have indicated a complicated role of miR-124a in pancreatic beta cell development and insulin production and secretion. One of the miR-124a target genes validated in pancreatic β cells is the transcription factor FOXA2 12, which regulates the expression of important β cell-specific genes such as PDX1 47, 48. It has also been reported that miR-124a inhibition increases the expression of NEUROD1 15, a transcription factor involved in insulin gene transcription 18, 49, and it has been proven that miR-124a can directly target neurod1 mRNA in the 3'-UTR in human cells 19. Using a miR-124a mimic transfection assay, we validated that miR-124a inhibited the expression of FOXA2 and NEUROD1 in MIN6 cells (Fig. 5A). However, although the EGFR inhibitor markedly blocked the expression of NEUROD1, EGF treatment did not result in a corresponding upregulation of this protein. Moreover, the level of FOXA2 was downregulated in the presence of EGF, while it was slightly increased under conditions of EGFR inhibitor or MAPK inhibitor treatment (Fig. 5A). Thus, our results show that EGF could not simply enhance the expression of miR-124a target genes via the downregulation of miR-124a, and clearly, EGF had a complex influence on beta cell function.

Since the effective distance of ETS2 to miR-124a was closer than that of EGF, we wondered whether ETS2 could regulate miR-124a target genes and then play a role in beta cell functions. We measured FOXA2 and NEUROD1 levels in ETS2 knockdown or ETS2-overexpressing MIN6 cells. As illustrated in Fig. 5B and 5C, FOXA2 and NEUROD1 expression was suppressed in ETS2-siRNA-transfected cells, while ETS2-overexpressing cells demonstrated a significant increase both at RNA and protein levels. Similar results were obtained in rat beta cells, INS-1 (Fig. S1C). Surprisingly, overexpression of the mutant type of ETS2 (when the phosphorylation site indispensable for the suppression of miR-124a expression was mutated) led to a similar upregulation in FOXA2 and NEUROD1 (Fig. 5C, S1C). To further determine if activation of Foxa2 and NeuroD1 by ETS2 was independent of miR-124a, we enforced miR-124a expression by using miR-124a mimics in ETS2-transfected cells. We found that excess miR-124a prevented the ETS2-mediated upregulation in NEUROD1, while it had little effect on the enhancement of FOXA2 expression induced by ETS2 (Fig. 5D). Therefore, ETS2 could regulate miR-124a target genes FOXA2 and NEUROD1, which may be partly through the inhibition of miR-124a expression.

### ETS2 has a positive role in insulin biosynthesis

**Figure 6:**
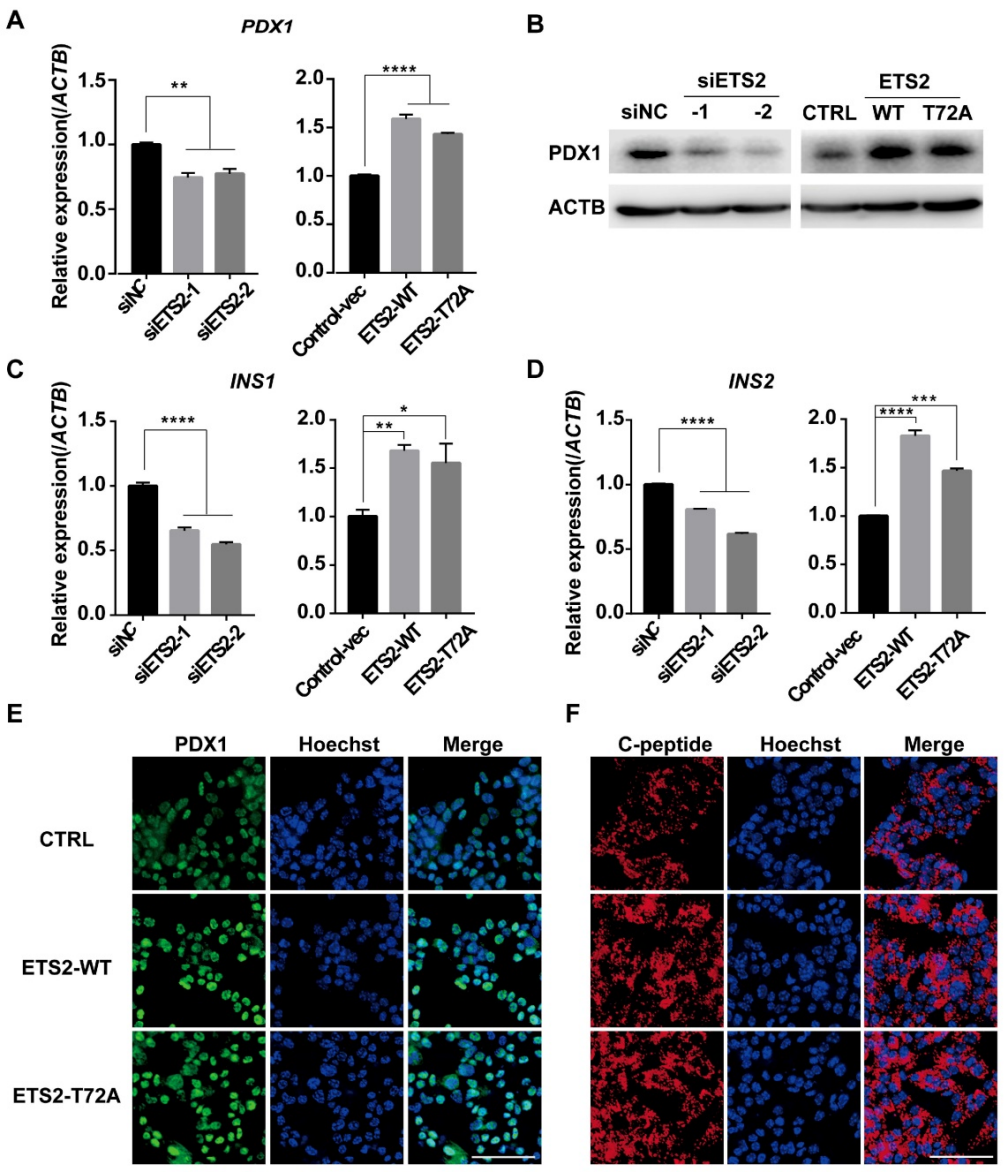
** ETS2 has a positive role in regulation of insulin synthesis key genes.** (A) Expression of *PDX1* was determined by qRT-PCR and in MIN6 cells transfected with siNC or siETS2, or control vector, ETS2-WT or ETS2-T72A overexpression plasmids. (B) Expression of *PDX1* was determined by western blot in ETS2-knockdown or overexpression cells. (C) Expression of *INS1* was determined by qRT-PCR in ETS2-Knockdown or overexpression cells. (D) The same as with (C), but for *INS2* expression detection. (E) Expression and location of PDX1 (green) were determined by immunofluorescence in MIN6 cells following control, ETS2-WT or ETS2-T72A overexpression plasmids transfection for 72h. Hoechst (blue), dye for nucleus staining. Scale bars, 50 μM. (F), the same with (E), but for C-peptide (red) staining.

**Figure 7:**
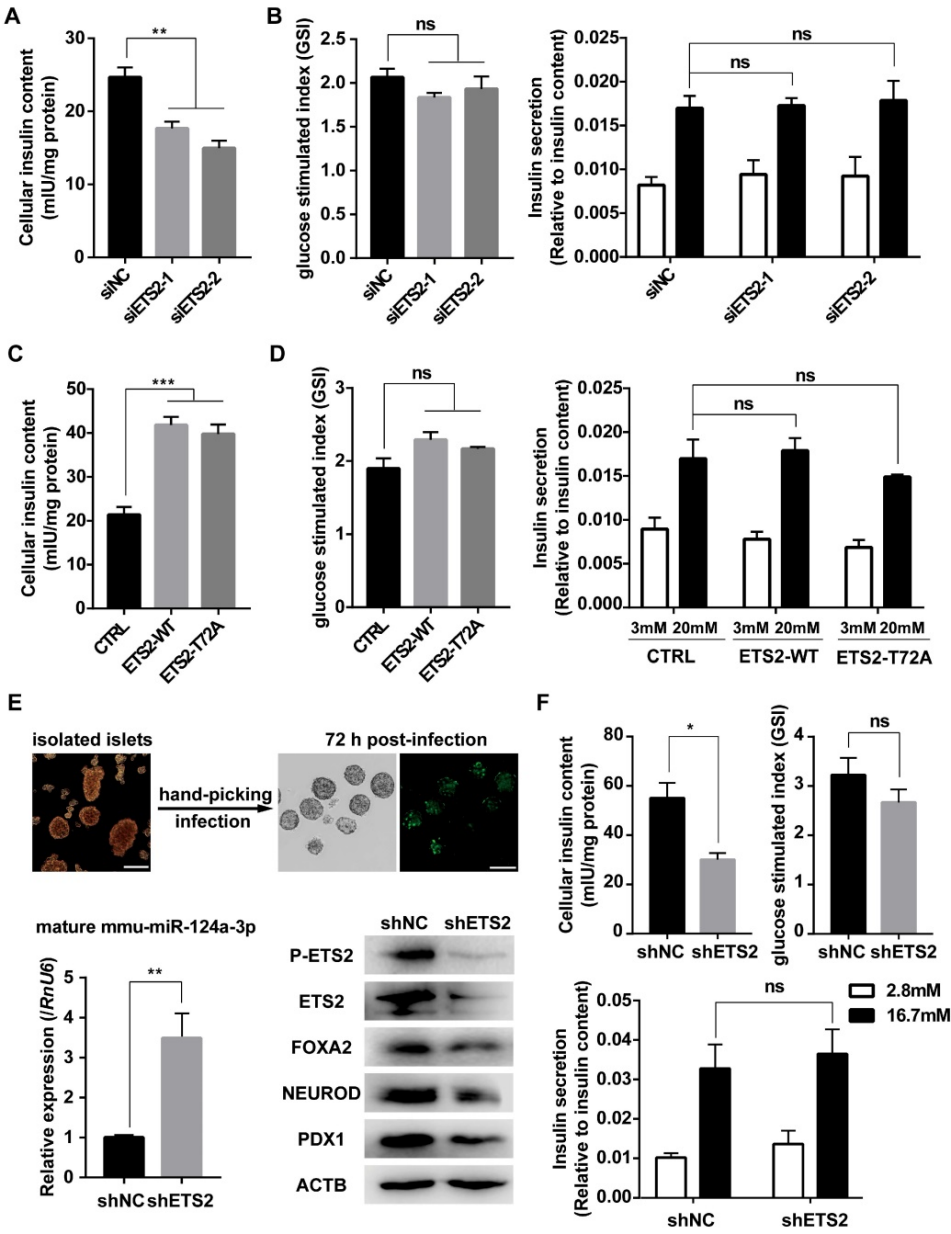
** ETS2 promotes insulin biosynthesis in insulinoma cells and isolated murine islets.** (A) ELISA was used to monitor the cellular insulin content in MIN6 cells following siNC or siETS2 transfection for 48 h. IU: International Unit. One unit of mouse insulin is contained in 0.0345 mg of the first International Standard. (B) ELISA was used to measure the glucose stimulated index (the ratio of the insulin value after high glucose stimulation divided by insulin value after low glucose stimulation) in MIN6 cells following indicated siRNA transfection for 48 h. And insulin secretion normalized to insulin content (3 mmol/l glucose, white bars; 20 mmol/l glucose, black bars) was measured. (C), the same in (A), but for cell transfected with control, ETS2-WT or ETS2-T72A overexpression plasmids. (D), the same in (B), but for cell transfected with control, ETS2-WT or ETS2-T72A overexpression plasmids. (E) Murine islets were isolated to confirm ETS2 function on miR-124a, FOXA2, NEUROD1 and PDX1 expression, after infection with pLKO.1-EGFP-shNC or shETS2 lentiviral particles for 72 h. Infection efficiency was visualized by EGFP fluorescence. Scale bars, 200 μM. (F) ELISA was used to monitor the cellular insulin content, glucose stimulated index, and insulin secretion normalized to insulin content (2.8 mmol/l glucose, white bars; 16,7 mmol/l glucose, black bars) in ETS2-knockdown or control islets. Values are means ± SE of three independent experiments (each n = 3). * P < 0.05, ** P < 0.01, *** P < 0.001, **** P < 0.0001. ns, not significant.

As a downstream gene activated by FOXA2, PDX1 was regulated by ETS2 (Fig. 6A, 6B, 6E and Fig. S1C). Importantly, PDX1 and NEUROD1 are known to play key roles in regulating insulin gene transcription 18, 48. As expected, similar effects on insulin mRNA level and protein content (represented by C-peptide) were also observed under conditions of ETS2 knockdown or overexpression (Fig. 6C, 6D, 6F, and Fig. S1C). ELISA for total cellular insulin content further illustrated the positive role of ETS2 in insulin biosynthesis (Fig. 7A and 7C). However, we did not find significant variation in the ability of insulin secretion (determined by glucose-stimulated index and the insulin secretion normalized to insulin content) after ETS2 silencing or overexpression (Fig. 7B and 7D). Although the level of insulin secreted from ETS2-silenced or overexpressed MIN6 cells was significantly varied in the presence of 20 mM glucose (Fig. S1D), the percentage of secreted insulin was similar to controls after standardizing with total insulin content (Fig. 7B and 7D). This indicated that, during glucose stimulated insulin secretion, the change of total insulin content in siETS2 or ETS2 transfected MIN6 cells was the major cause of varied insulin release. We also verified the function of ETS2 in isolated mouse islets. As showed in Fig. 7E, knockdown of ETS2 by lentivirus infection led to elevated miR-124 expression, following decreased expression of insulin biosynthesis related genes such as FOXA2, NEUROD1, and PDX1. Impaired insulin biosynthesis and unaffected insulin secretion were also observed in shETS2-islets (Fig. 7F, S1E). These were consistent to the results in beta cell lines.

## Discussion

Studies have shown that miR-124a plays an important role in pancreatic endocrine development 13, 37, insulin biosynthesis and secretion, and insulin signal transduction 12, 15. Despite these important biological roles, little is known about the regulation of miR-124 biogenesis in the pancreas. Our previous study showed a mutual inhibition between EGF and miR-124a to determine the proliferation and differentiation of pancreatic progenitor cells 37, while in differentiated beta cells, it was unknown whether EGFR contributes to control miR-124 expression to regulate beta cell function. We described here that EGF activated EGFR signaling cascades, including the MAPK and PI3K pathways, to down-regulate the expression of miR-124a in MIN6 and INS-1 cells (Fig. 1 and 2). However, we did not detect a consistent positive effect of EGFR signaling on miR-124a targets (FOXA2 and NEUROD1) according to EGF and EGFR inhibitor treatments (Fig. 5A). Considering the multiplicity of interactions between microRNA and its targets and the complexity of effects of EGFR signaling on cell survival and proliferation, EGFR signaling-mediated inhibition of miR-124a expression may comprehensively function to regulate the proper activity of beta cells.

ETS2 is a member of the ETS (E26 transformation-specific sequence) family of transcription factors that participates in cellular proliferation, differentiation and apoptosis, thus playing important roles in embryo development 50-53, senescence 54, vasculogenesis 55, immunity 56-59, osteogenesis 60, adipogenesis 61, and tumorigenesis 62, 63. Some members of the ETS family, such as Etv4 and Etv5, have been indicated in pancreatic development mediating mesenchymal-to-epithelial signaling 64. ELK1 65 and ETS1 66-68 have been reported to inhibit glucose-stimulated insulin secretion by pancreatic β cells and to be related to beta cell dysfunction. Compared with other ETS family members, ETS2 was the most abundant factor expressed during pancreatic morphogenesis 64, as well as in normal adult pancreatic islets (data not shown), which indicated the vital role of ETS2 in pancreatic development and function 64; however, the relevant details have not yet been addressed. In this study, we showed that ETS2 inhibited miR-124a expression at the transcriptional level and in an ERK1/2- or/and PI3K-dependent manner. We also demonstrated that ETS2 has a positive role in insulin biosynthesis in pancreatic β cells.

ETS2 is an important substrate of the MAPKs ERK1/2 or PI3K, which can be activated by growth factors or cellular stress 43, 44, 69-71. In our study, we determined that both the ERK1/2 and PI3K signaling pathways are involved in the stimulation of ETS2 phosphorylation in MIN6 cells, since the ERK1/2 or PI3K inhibitor alone could decrease the EGF-induced phosphorylation of ETS2 at threonine 72 (Fig. 2). Here, we found that the suppression of ETS2 activity attenuated EGF-mediated regulation of miR-124a expression by siETS2 or inhibitors of ERK1/2 or PI3K (Fig. 2D and Fig. 3D, 3E), supporting the notion that ETS2 acts as a downstream effector of the EGF/Ras/ERK or EGF/PI3K/AKT pathway to repress miR-124a expression in pancreatic β cells. Phosphorylation at threonine 72 is important for ETS2 activation and has been well studied 71, and it was also found to be essential for ETS2 function in miR-124a regulation (Fig. 3C), and this phenomenon was also found to be essential for ETS2 functions in miR-124a regulation (Fig. 3C). However, ETS2 is phosphorylated on other residues through the ERK or AKT pathway, and the role(s) that these other sites play in the regulation of miR-124a expression remains to be determined. ETS2 has been reported to control the transcription of miRNAs. ETS2 regulates microRNA-126 in endothelial cells 72, stimulates miR-155 during the inflammatory response 73, and represses the expression of miR-196b in gastric cancer cells 74 by binding to motifs containing the conserved sequence GGAA on the promoter. A similar mechanism was identified for the regulatory effect of ETS2 on the miR-124 promoter, and the core conserved sequence AAGGAANA/TN was found to be indispensable in this process (Fig. 4E-4G). Luciferase assay results showed that ETS2 displayed a strong suppressive ability on the miR-124a3 promoter, which may be the result of multiple binding sites located in this region (Fig. 4E, 4F). We identified 100% identical evolutionarily conserved binding motifs between mice and rats (Fig. S1B), in accordance with the similar inhibition of miR-124a expression by EGFR signaling (Fig. 1), further indicating a transrepression role of ETS2 in miR-124a regulation. Nevertheless, it remains to be investigated whether the repressor role of ETS2 in the regulation of miR-124a is dependent on other posttranslational modifications or specific binding partners, as described in 75 that ETS2 can switch between being an activator and being a suppressor by specifically interacting with the co-repressor ZMYND11.

We next found that ETS2 has a positive effect on the transcription factors FOXA2, NEUROD1 and PDX1 (Fig. 5B-5D and Fig. 6), which are known to not only control beta cell development but also maintain mature beta cell function 18, 47-49, 76-79. NEUROD1, which has been reported to modulate insulin expression, has been confirmed to be regulated by miR-124a (Fig. 5A). Studies have shown that miR-124a targets FOXA2 expression, consequently regulating PDX1 and pre-proinsulin genes (*INS1*, *INS2*) transcription 12. We demonstrated that ETS2-silencing induced a decrease in FOXA2, NEUROD1, PDX1, INS1 and INS2 expression. A reverse effect on these genes was observed in response to ETS2 overexpression (Figs. 5 and 6), potentially reflecting a decrease in miR-124a level. However, mutant ETS2 without the phosphorylation site (T was mutated to A), which has been shown disappeared inhibition on miR-124a expression (Fig. 2C), still participated in the positive regulation of these genes. Thus, ETS2 might regulate NEUROD1 and FOXA2 partly or completely independent of miR-124a. Paradoxically, restored miR-124a eliminated ETS2-overexpression-induced promotion of NEUROD1 expression (Fig. 5D). We suggested that it might result from faster speed of mRNA degradation by excess exogenous miR-124a than that of mRNA production activated by EST2. For FOXA2, results showed that ETS2 could promote FOXA2 expression at mRNA levels (Fig. 5B, 5C, 5D), while miR-124a mimics slightly reduced FOXA2 mRNA levels, but significantly decreased its protein levels in cells without ETS2 overexpression, which was consistent with the previous report 12, indicating translational regulation role of miR-124a on FOXA2 (Fig. 5A, 5D). Therefore, even excess exogenous miR-124a could not block the transcriptional activation of FOXA2 by ETS2. And these results indicate that there exist other pathways or mechanisms by which ETS2 regulates FOXA2 and NEUROD genes independent of miR-124a. Changes in PDX1 and INS1 and INS2 expression may result from variations in FOXA2 and NEUROD and/or direct control of ETS2, following corresponding variation in insulin production both in beta cell lines and isolated mouse islets (Fig. 6 and 7), which was consistent with PDX1 and insulin expression under the same conditions. No change was apparent in ability of glucose-induced insulin secretion after ETS2 treatment (Fig. 6, 7B, 7D and 7F). Thus, ETS2 may not influence the final step of the secretory pathways such as molecular machinery that drives insulin exocytosis, although miR-124a has been reported to impair insulin secretion 15, 17. There may be homeostatic mechanisms for ETS2 to avoid excess insulin secretion by antagonizing its inhibition on miR-124a, thus maintaining a proper beta cell function.

## Conclusions

**Figure 8:**
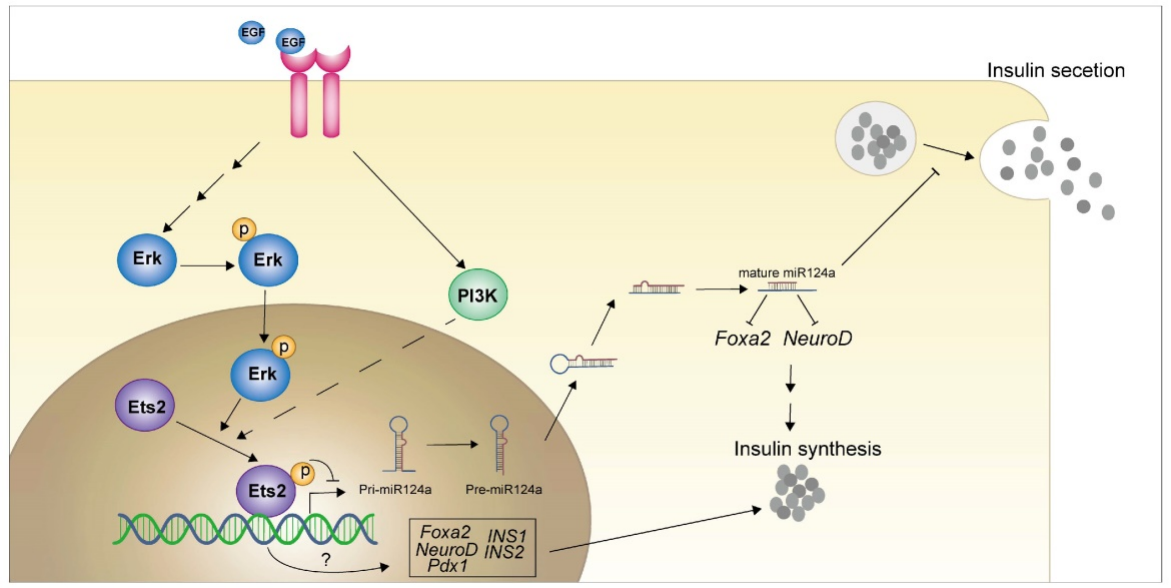
Graphical abstract of how EGFR signaling pathway and downstream effector inhibit the miR-124a expression and the role of ETS2 in insulin production in pancreatic beta cells.

In present study, we showed that EGF activated MEK/ERK and PI3K/AKT signaling cascades to down-regulate miR-124a expression, and a member of ETS family of transcription factors, ETS2, acted as the downstream effector of EGFR signaling to suppression the promoter activity of miR-124a (a kind of miRNA that related to beta cell dysfunction in diabetes). Moreover ETS2 was discovered to positively regulate insulin biogenesis related genes *FOXA2*, *NEUROD1*, *PDX1*, *INS1* and *INS2* in a T72 phosphorylation independent manner (Fig. 8). Our results expand our understanding of mechanisms of inappropriate regulation of miR-124a involved in processes leading to human disease such as diabetes mellitus, and imply a series of new ETS2 functions, and support future work in exploring this pathway in vivo.

## Supplementary Material

Supplementary figure and tables.
