# Supplementary material for: EGF suppresses the expression of miR-124a in pancreatic β cell lines via ETS2 activation through the MEK and PI3K signaling pathways
Source: Int J Biol Sci. 2019 Sep 7;15(12):2561–75. doi: 10.7150/ijbs.34985 (PMC6854373; doi:10.7150/ijbs.34985)
Supplement: Supplementary file 1 — Supplementary figure and tables. [file ijbsv15p2561s1.pdf]

Supplementary table 1:

Primers for mature miR-124a and pre-miR-124a quantification

Name	sequence
**mature miR-124a**	GTCGTATCCAGTGCGTGTCGTGGAGTCGGCAATT
**reverse-transcription primer**	GCACTGGATACGACGGCATTC
**mmu/Rno-miR-124a-Forward**	TTTGGTAAGGCACGCGGT
**mmu/Rno-miR-124a-Reverse**	CAGTGCGTGTCGTGGAGT
**mmu/Rno-RnU6-Forward**	CTCGCTTCGGCAGCACA
**mmu/Rno-RnU6-Reverse**	AACGCTTCACGAATTTGCGT
**mmu-pre-mir124-all-Forward**	TCCGTGTTCACAGCGGAC
**mmu-pre-mir124-all-Reverse**	CATTCACCGCGTGCCTTA

Supplementary table 2:

Primers for Real-time quantitative PCR

Name	sequence
**mmu-ETS2-Forward**	GAAAACTCTCACCTCAACGC
**mmu-ETS2-Reverse**	ACATGCTGTCCAGGAGATTG
**mmu-ACTB-Forward**	CAAGCAGGAGTACGATGAGTC
**mmu-ACTB-Reverse**	AACGCAGCTCAGTAACAGTC
**mmu-FOXA2-Forward**	GCAGACACTTCCTACTACCAAG
**mmu-FOXA2-Reverse**	TTCCTCAAAGCTCTCCCAAAG
**mmu-neurod1-Forward**	CTCCAGGGTTATGAGATCGTC
**mmu-neurod1-Reverse**	GTCCTGAGAACTGAGACACTC
**mmu-PDX1-Forward**	CCCTTTCCCGTGGATGAAATC
**mmu-PDX1-Reverse**	GAATTCCTTCTCCAGCTCCAG
**mmu-INS1-Forward**	ATCAGAGACCATCAGCAAGC
**mmu-INS1-Reverse**	GTTTGACAAAAGCCTGGGTG
**mmu-INS2-Forward**	GGCTTCTTCTACACACCCATG
**mmu-INS2-Reverse**	TGATCTACAATGCCACGCTTC
**Rno-FOXA2-Forward**	CTGGGACTTAACTGTAACGGG
**Rno-FOXA2-Reverse**	ATGTTGCTCACGGAAGAGTAG
**Rno-neurod1-Forward**	TCATGAGTGCCCAGCTTAATG
**Rno-neurod1-Reverse**	ACAGTGGATTCGTTTCCCG
**Rno-PDX1-Forward**	GGCTTAACCTAAACGCCACA
**Rno-PDX1-Reverse**	AGAGTCCCAGAGGCAGACCT
**Rno-INS1-Forward**	ATCTTCAGACCTTGGCACTG
**Rno-INS1-Reverse**	GGCTTTATTCATTGCAGAGGG
**Rno-INS2-Forward**	CCCAGGCTTTTGTCAAACAG
**Rno-INS2-Reverse**	CACCCAGCTCCAGTTGTG
**Rno-ACTB-Forward**	CAAGCAGGAGTACGATGAGTC
**Rno-ACTB-Reverse**	AACGCAGCTCAGTAACAGTC

Supplementary table 3:

Primers for plasmid construction

Vector name	Direction	Sequence
**pCDNA3.1-ETS2**	Forward	gtgcccgattacgctggatccGCCACCATGAATGACTTTGGAATCAAGAACA
	Reverse	tgctggatatctgcagaattcGTCTTCTGTATCAGGCTGGACGC
**pCDNA3.1-ETS2-T72A**	Forward	gtgcccgattacgctggatccGCCACCATGAATGACTTTGGAATCAAGAACA
	Reverse	tgctgcagggAGcGAGCAAAGGCAGCTCGC
	Forward	tttgctcgctCCCTGCAGCAAGGCAGTG
	Reverse	tgctggatatctgcagaattcGTCTTCTGTATCAGGCTGGACGC
**pGL3-miR124a1-promoter**	Forward	atctgcgatctaagtaagcttGGAAGCACTTGGGGCTTGG
	Reverse	cagtaccggaatgccaagcttTTCTCCTGAGGAAGGAAGAAGGA
**pGL3-miR124a2-promoter**	Forward	atctgcgatctaagtaagcttTAGGTAGTTGTATTGGGGCCAGTG
	Reverse	cagtaccggaatgccaagcttCTAGAAGCAAGTGACTCATTGCAAC
**pGL3-miR124a3-promoter**	Forward	atctgcgatctaagtaagcttGACTGGCACCTGCAAAGGG
	Reverse	cagtaccggaatgccaagcttGGGCCCTCAGGGCTGCGG
**pGL3-mut-miR124a1-promoter**	Forward	atctgcgatctaagtaagcttGGAAGCACTTGGGGCTTGG
	Reverse	ttccaagcttGCAAAGGGGAGGGGGGTG
	Forward	tcccctttgcAAGCTTGGAAACGAAAGGATGC
	Reverse	cagtaccggaatgccaagcttTTCTCCTGAGGAAGGAAGAAGGA
**pGL3-mut-miR124a2-promoter**	Forward	atctgcgatctaagtaagcttTAGGTAGTTGTATTGGGGCCAGTG
	Reverse	caggacgagagaaatgtGAGATGGAGTCTTGAAAGCGAGTC
	Forward	ctcACATTTCTCTCGTCCTGACTGCA
	Reverse	cagtaccggaatgccaagcttCTAGAAGCAAGTGACTCATTGCAAC
**pGL3-mut-miR124a3-promoter**	Forward	atctgcgatctaagtaagcttGACTGGCACCTGCAAAGGG
	Reverse	ttgGCAGACTGGAAGAACCACACAGA
	Forward	tggttcttccagtctgcCAAGGCCCGCTGGGAAGG
	Reverse	aaggaagccgggcaaGAGGGCCGGGTGGGAGCC
	Forward	ccctcTTGCCCGGCTTCCTTGGC
	Reverse	aactccgaggagccagtTCCCCTCCCCGAGGTAGAG
	Forward	ggaACTGGCTCCTCGGAGTTGGG
	Reverse	cagtaccggaatgccaagcttGGGCCCTCAGGGCTGCGG
**pLKO.1-EGFP-Puro-shETS2-3153**	Forward	CCGGCATTGATAAAGAGCCGTTATACTCGAGTATAACGGCTCTTTATCAATGTTTTTG
	Reverse	AATTCAAAAACATTGATAAAGAGCCGTTATACTCGAGTATAACGGCTCTTTATCAATG

Supplementary table 4:

Sequences of siRNA and miRNA mimics

Name	sequence

**MiR-124a mimics sense**	UAAGGCACGCGGUGAAUGCC
**MiR-124a mimics antisense**	AUUCCGUGCGCCACUUACGG
**SiETS2-1-987 sense**	CAACACCGUCAAUGUCAAUUA
**SiETS2-1 antisense**	UAAUUGACAUUGACGGUGUUG
**SiETS2-2-3153 sense**	CAUUGAUAAAGAGCCGUUAUA
**SiETS2-2 antisense**	UAUAACGGCUCUUUAUCAAUG
**NC /siNC sense**	UUCUCCGAACGUGUCACGU
**NC /siNC antisense**	ACGUGACACGUUCGGAGAA

Supplementary figure 1

Figure S1

(A) Primers used for detecting miR-124a isoforms simultaneously are indicated. The sequences of the mouse *miR-124a* precursors are shown. Conserved nucleotides among isoforms are shaded.

(B) Conserved ETS2 binding sites (EBS) on miR-124a promoters between mouse and rat are represented. Promoters (-2000 ~ +1) from the mouse and rat miR-124a genomic regions at indicated location, are aligned, and the conserved EBS sequences and location on corresponding promoters are indicated.

(C) Expression of FOXA2, NEUROD1, PDX1, INS1 and INS2 was determined by qRT-PCR and western bolts in INS-1 cells transfected with control, ETS2-WT or ETS2-T72A overexpression plasmids. Values are means ± SE of three independent experiments (each n = 3). * $P < 0.05$, ** $P < 0.01$, *** $P < 0.001$, **** $P < 0.0001$. ns, not significant.

(D) ELISA was used to monitor the released insulin levels (normalized to protein level) in ETS2 silencing or overexpression MIN6 cells.

(E), the same with (D), but in ETS2-knockdown or control islets.

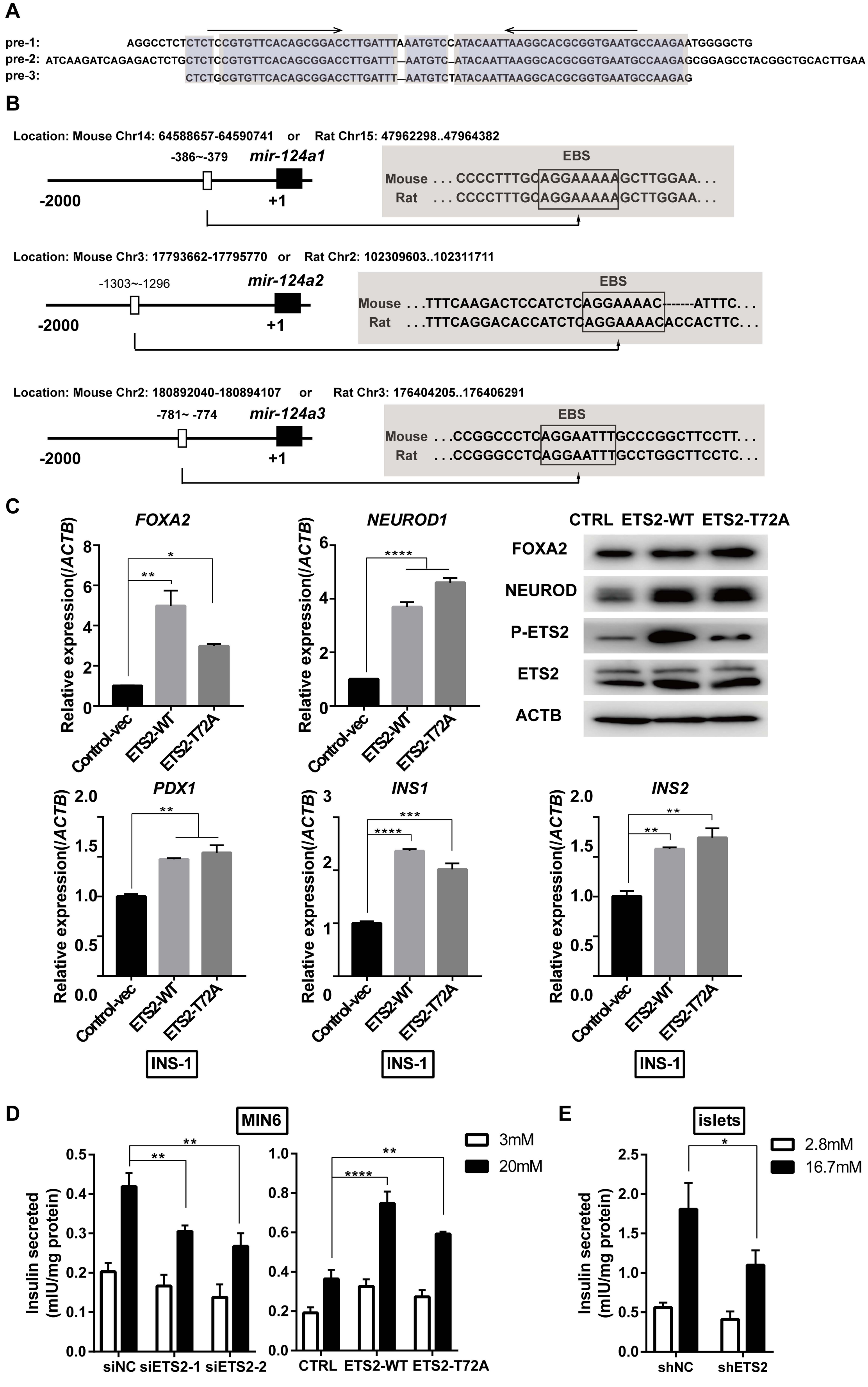

A
pre-1: AGGCCTCTCTCTCCGTGTTCACAGCGGACCTTGATTTAAATGTCCATACAATTAAGGCACGCGGTGAATGCCAAGAATGGGGCTG
pre-2: ATCAAGATCAGAGACTCTGCTCTCCGTGTTCACAGCGGACCTTGATTT—AATGTC—ATACAATTAAGGCACGCGGTGAATGCCAAGAGCGGAGCCTACGGCTGCACTTGAA
pre-3: CTCTGCGTGTTCACAGCGGACCTTGATTT—AATGTCTATACAATTAAGGCACGCGGTGAATGCCAAGAG
B
Location: Mouse Chr14: 64588657-64590741 or Rat Chr15: 47962298..47964382
-386~-379
mir-124a1
-2000
+1
EBS
Mouse . . . CCCCTTTGCAGGAAAAAGCTTGGAA. . .
Rat . . . CCCCTTTGCAGGAAAAAGCTTGGAA. . .
Location: Mouse Chr3: 17793662-17795770 or Rat Chr2: 102309603..102311711
-1303~-1296
mir-124a2
-2000
+1
EBS
Mouse . . .TTTCAAGACTCCATCTCAGGAAAAC-------ATTTC. . .
Rat . . .TTTCAGGACACCATCTCAGGAAAACACCACTTC. . .
Location: Mouse Chr2: 180892040-180894107 or Rat Chr3: 176404205..176406291
-781~ -774
mir-124a3
-2000
+1
EBS
Mouse . . .CCGGCCCTCAGGAATTTGCCCGGCTTCCTT. . .
Rat . . .CCGGGCCTCAGGAATTTGCCTGGCTTCCTC. . .
C
FOXA2
NEUROD1
Relative expression(/ACTB)
Control-vec
ETS2-WT
ETS2-T72A
CTRL ETS2-WT ETS2-T72A
FOXA2
NEUROD
P-ETS2
ETS2
ACTB
PDX1
INS1
INS2
INS-1
D
MIN6
3mM
20mM
Insulin secreted (mIU/mg protein)
siNC siETS2-1 siETS2-2
CTRL ETS2-WT ETS2-T72A
E
islets
2.8mM
16.7mM
shNC
shETS2
